# The Small Regulatory Antisense RNA PilR Affects Pilus Formation and Cell Motility by Negatively Regulating *pilA11* in *Synechocystis* sp. PCC 6803

**DOI:** 10.3389/fmicb.2018.00786

**Published:** 2018-04-23

**Authors:** Jinlu Hu, Jiao Zhan, Hui Chen, Chenliu He, Huaixing Cang, Qiang Wang

**Affiliations:** ^1^School of Life Sciences, Northwestern Polytechnical University, Xi'an, China; ^2^Key Laboratory of Algal Biology, Institute of Hydrobiology, Chinese Academy of Sciences, Wuhan, China; ^3^Donghu Experimental Station of Lake Ecosystems, State Key Laboratory of Freshwater Ecology and Biotechnology of China, Institute of Hydrobiology, Chinese Academy of Sciences, Wuhan, China

**Keywords:** *Synechocystis* sp. PCC 6803, PilR, *pilA11*, pili, cell motility

## Abstract

Pili are found on the surface of many bacteria and play important roles in cell motility, pathogenesis, biofilm formation, and sensing and reacting to environmental changes. Cell motility in the model cyanobacterium *Synechocystis* sp. PCC 6803 relies on expression of the putative *pilA9-pilA10-pilA11-slr2018* operon. In this study, we identified the antisense RNA PilR encoded in the noncoding strand of the prepilin-encoding gene *pilA11*. Analysis of overexpressor [PilR(+)] and suppressor [PilR(−)] mutant strains revealed that PilR is a direct negative regulator of PilA11 protein. Although overexpression of PilR did not affect cell growth, it greatly reduced levels of *pilA11* mRNA and protein and decreased both the thickness and number of pili, resulting in limited cell motility and small, distinct colonies. Suppression of PilR had the opposite effect. A hypothetical model on the regulation of *pilA9-pilA10-pilA11-slr2018* operon expression by PilR was proposed. These results add a layer of complexity to the mechanisms controlling *pilA11* gene expression and cell motility, and provide novel insights into how sRNA and the intergenic region secondary structures can work together to discoordinatly regulate target gene in an operon in cyanobacterium.

## Introduction

Cyanobacteria are ancient organisms that perform oxygenic photosynthesis (Waterbury et al., 1985). According to endosymbiotic theory, plant chloroplasts originated from cyanobacteria (or a cyanobacteria-like organism) through primary endosymbiosis. Many cyanobacteria move by gliding, swimming, or twitching (Waterbury et al., 1985; Häder, 1987). Gliding motility is a slow, uniform, forward motion, which parallel to the cell's longitudinal axis on a solid surface (Häder, 1987). This type of motion is occasionally interrupted by reversals in filamentous cyanobacteria such as *Phormidium uncinatum* and *Anabaena variabilis* (Häder, 1987). Several marine species of unicellular *Synechococcus* show swimming motility through liquids at a rate of 25 μm s^−1^(Waterbury et al., 1985). Twitching motility is small and intermittent translocation on a solid surface with frequent changes in direction (Henrichsen, 1972). *Synechocystis* sp. PCC 6803, a model unicellular cyanobacterium, exhibits twitching motility on an agar plate or glass slide (Stanier et al., 1971; Ng et al., 2003).

The pilus is a hair-like appendage found on the surface of many single-celled prokaryotes and has emerged as an efficient device for cell motility, pathogenesis (Herrington et al., 1988; Strom and Lory, 1993; Sauer et al., 2000), biofilm formation (Pratt and Kolter, 1998; Barken et al., 2008), and environmental sensing (Kawagishi et al., 1996). The genomic sequencing of *Synechocystis* sp. PCC 6803 was finished in 1996 (Kaneko et al., 1996). Since then, a number of genes (known as the *pil* genes) involved in pilus biogenesis, cell motility, and transformation competency have been revealed by mutational analysis (Bhaya et al., 1999, 2000, 2001; Yoshihara et al., 2001), which show homology to type IV pili biogenesis genes in many Gram-negative bacteria. Nonflagellar appendages of Gram-negative bacteria can be categorized into five major classes based on their biosynthetic pathway (Fronzes et al., 2008; Lo et al., 2013): chaperone–usher pili (Sauer et al., 1999; Waksman and Hultgren, 2009; Busch and Waksman, 2012; Geibel and Waksman, 2014; Pham et al., 2016), curli (Olsén et al., 1989; Barnhart and Chapman, 2006; Green et al., 2016), type IV pili (Bhaya et al., 2000; Merz et al., 2000; Maier et al., 2002; Busch and Waksman, 2012; Busch et al., 2015), type III secretion needle (Roine et al., 1997; Kubori et al., 1998), and type IV secretion pili (Seubert et al., 2003; Schröder and Lanka, 2005). Eleven *pilA*-like genes are contained in *Synechocystis* genome, which encode a prepilin peptide with a characteristic sequence (Yoshihara et al., 2002; Yoshimura et al., 2002). Of these genes, *pilA10, pilA11*, and *slr2018* function in cell motility (Bhaya et al., 2001), and *pilA1* is essential for the formation of thick and thin pili (Bhaya et al., 2000; Yoshihara et al., 2001).

Transcriptome analyses have identified numerous noncoding transcripts in bacteria, mainly trans-encoded RNAs and cis-antisense RNAs (asRNAs) (Waters and Storz, 2009). Cis-encoded asRNA transcripts appear to be dominant in several cyanobacteria. For example, asRNAs respectively comprise 26 and 39% of all genes in *Synechocystis* sp. PCC 6803 (Georg et al., 2009; Mitschke et al., 2011a) and *Anabaena* sp. PCC 7120 (Mitschke et al., 2011b). Chromosomally encoded asRNAs may play important roles in the regulatory networks of cyanobacteria. During the past decade, numerous newly discovered asRNAs have been shown to be involved in a wide range of processes (Kopf and Hess, 2015), including stress responses, photoprotection, low carbon responses, and carbon assimilation (Dühring et al., 2006; Eisenhut et al., 2012; Sakurai et al., 2012; Hu et al., 2017).

The transcriptome analysis using differential RNA sequencing in *Synechocystis* sp. PCC 6803 (Xu et al., 2014; Hu et al., 2017) revealed a low-abundance asRNA encoded in the noncoding strand of *pilA11*, which was named PilR. In this study, we determined the molecular functions of PilR by identifying its target gene, *pilA11*. An analysis of mutants with either elevated or reduced levels of PilR expression showed that PilR plays a key role in pilus formation and cell motility by negatively regulating *pilA11* expression.

## Results

### Characterization of PilR

**Figure 1:**
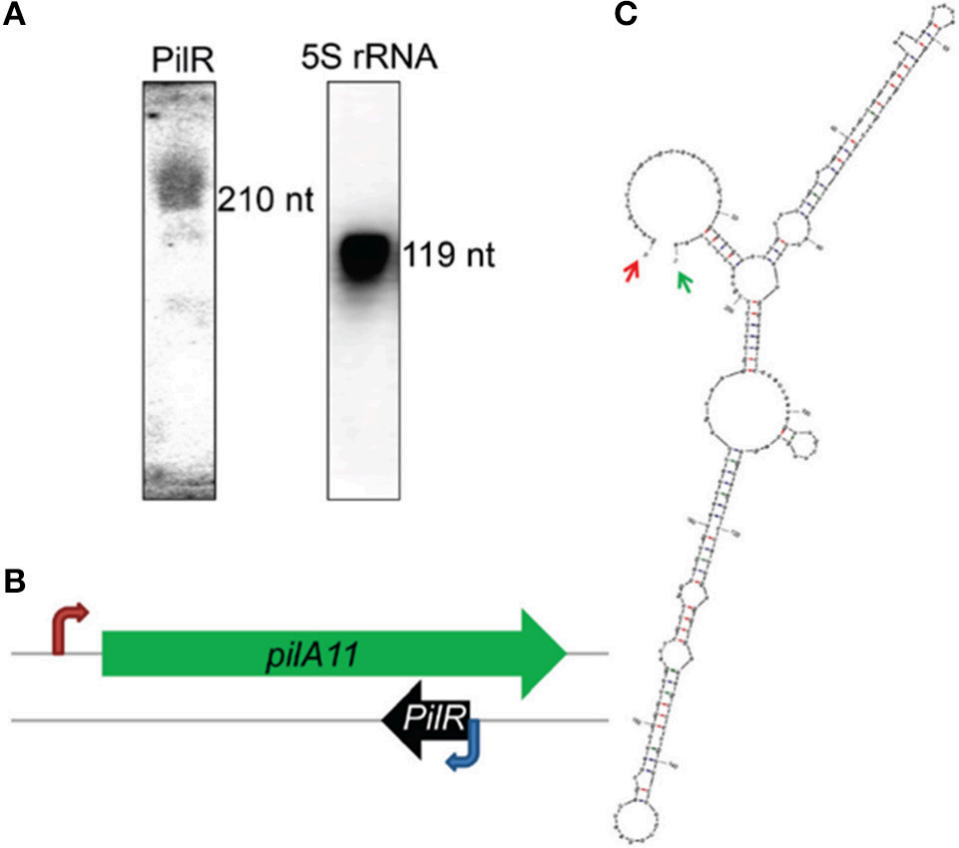
Characterization of the antisense RNA PilR in *Synechocystis* sp. PCC 6803. **(A)** Typical detection pattern of PilR and 5S rRNA by Northern blot analysis. Five micrograms of total RNA were loaded for detection of 5S rRNA. Fifty micrograms of total RNA were used to detect PilR. **(B)** Location of the *pilA11* gene within the genome. The red arrow marks the transcription initiation site. The blue arrow marks the PilR transcription initiation site detected by the RACE experiments. **(C)** The RNA secondary structure prediction for PilR. Arrows point to the experimentally detected 5′ (red) and 3′ (green) ends.

Differential RNA sequencing of the *Synechocystis* sp. PCC 6803 (hereafter *Synechocystis*) transcriptome detected an asRNA, designated PilR, with a transcription start site at the 3′ end of the *pilA11* gene, but on its complementary strand. PilR was determined to be 210 long using Northern blot (Figure 1A) and RACE analyses, and its transcription start site was mapped to nucleotide nt758305 in the sequenced genome. PilR extends from positions 677–886 in the coding sequence of *pilA11* (Figure 1B), and can be folded into two extended stem regions, with a terminal loop at each ending as predicted by the mfold software (http://www.bioinfo.rpi.edu/applications/mfold/; Figure 1C), such loops structures are believed to be involved in RNA–RNA interactions and therefore functionally related to the hypothetical trans-acting function (Dühring et al., 2006).

**Figure 2:**
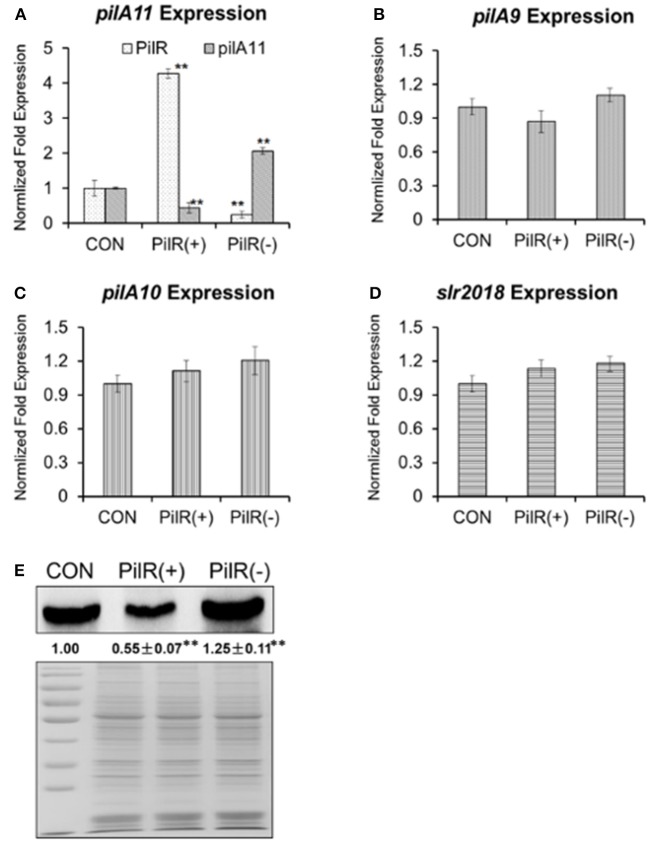
The influence of PilR on *pilA9–pilA10–pilA11–slr2018* operon transcription and PilA11 protein. The expression levels of **(A)** PilR (white) and *pilA11* mRNA (gray), **(B)** *pilA9* mRNA (gray), **(C)** *pilA10* mRNA (gray), and **(D)** *slr2018* mRNA (gray) in cells with normal (control), overexpressed [PilR(+)], and suppressed [PilR(-)] levels of PilR in BG11 medium as measured by qRT–PCR. All data are shown as means ± SD (*n* = 5). **(E)** Immunoblot of PilA11 protein isolated from total protein of control, PilR(+), and PiIR(−) mutant cells in BG11 medium. Significant differences between the control (CON) and test values were determined using a one–way ANOVA. ^**^P < 0.01 vs. CON.

We further constructed background control, PilR overexpression [PilR(+)], and PilR suppression [PilR(−)] strains (Supplementary Figure S1) for investigating the relationship between PilR and its target gene, *pilA11*. PilR appears to be a negative regulator of *pilA11* expression during the exponential growth phase, as revealed through qRT-PCR and immunoblot analyses of these strains (Figures 2A,E). As shown in Figure 2A, the levels of asRNA PilR were 4.28-fold higher in the PilR(+) strain than in the control, and 0.25-fold lower in the PilR(−) strain than in the control (Figure 2A, white speckled bars; ^**^P < 0.01). By contrast, the levels of *pilA11* mRNA were 0.45-fold lower in the PilR(+) strain than in the control, and 2.06-fold higher in the PilR(−) strain than in the control (Figure 2A, gray bars, ^**^P < 0.01). Similarly, the levels of PilA11 protein were 0.55-fold lower in the PilR(+) strain than in the control, and 1.25-fold higher in the PilR(−) strains than in the control (Figure 2E).

As previous investigations on the expression levels of the six genes (*slr1667, slr1668, pilA9, pilA10, pilA11*, and *slr2018*) strongly suggested that *pilA9, pilA10, pilA11*, and *slr2018* constitute one operon (Kamei et al., 2001a; Yoshimura et al., 2002; Panichkin et al., 2006) to verify that PilR affects *pilA11* but no other genes in the *pilA9*-*pilA10*-*pilA11-slr2018* operon, *pilA9, pilA10*, and *slr2018* transcript levels were also analyzed by qRT-PCR in the mutant strains (Figures 2B–D). We found that the mRNA levels of *pilA9* were 0.87-fold lower in the PilA(+) strain than in the control, and 1.10-fold higher in the PilA(−) strain (Figure 2B, *P* > 0.05); those of *pilA10* were 1.11-fold higher in the PilA(+) strain than in the control, and 1.20-fold higher in the PilA(−) strain (Figure 2C, *P* > 0.05); and those of *slr2018* were 1.13-fold higher in the PilA(+) strain than in the control, and 1.18-fold higher in the PilA(−) strain (Figure 2D, *P* > 0.05). These results suggest that PilR has no significant effect on the stability of the mRNA portions that encode *pilA9, pilA10*, and *slr2018*, providing strong evidence that PilR, despite its relatively low steady-state expression level, negatively regulates the amount of *pilA11* mRNA and PilA11 protein in *Synechocystis*.

### PilA11 localization

**Figure 3:**
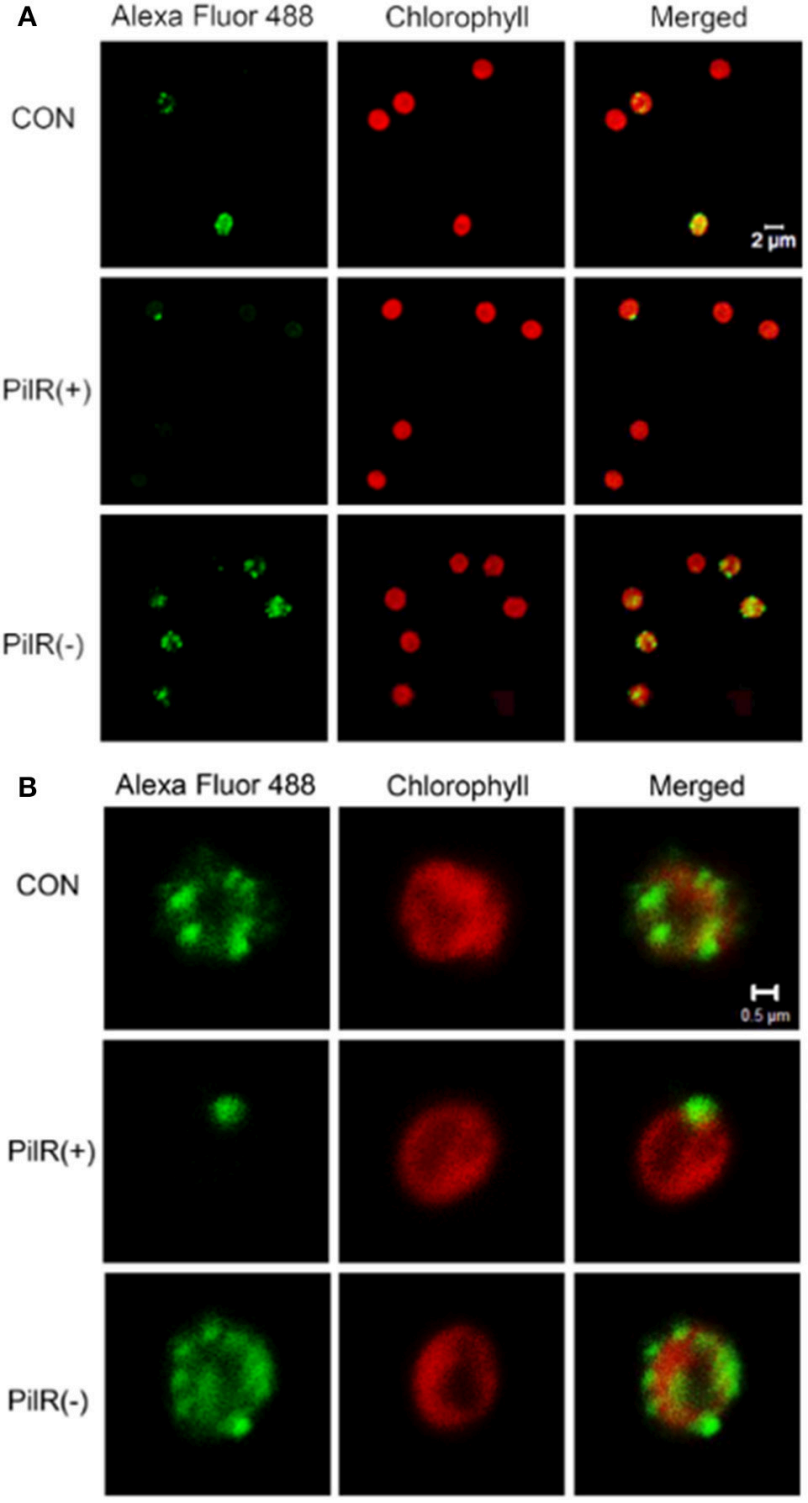
Fluorescence images showing the control, PilR(+), and PiIR(−) cells labeled with PilA11–Alexa Fluor 488. **(A)** A group of individual cells. **(B)** A single cell. The merged panel shows images of cells labeled with PilA11 (green) and chlorophyII (red) merged together. White bars = 2 and 0.5 μm.

**Figure 4:**
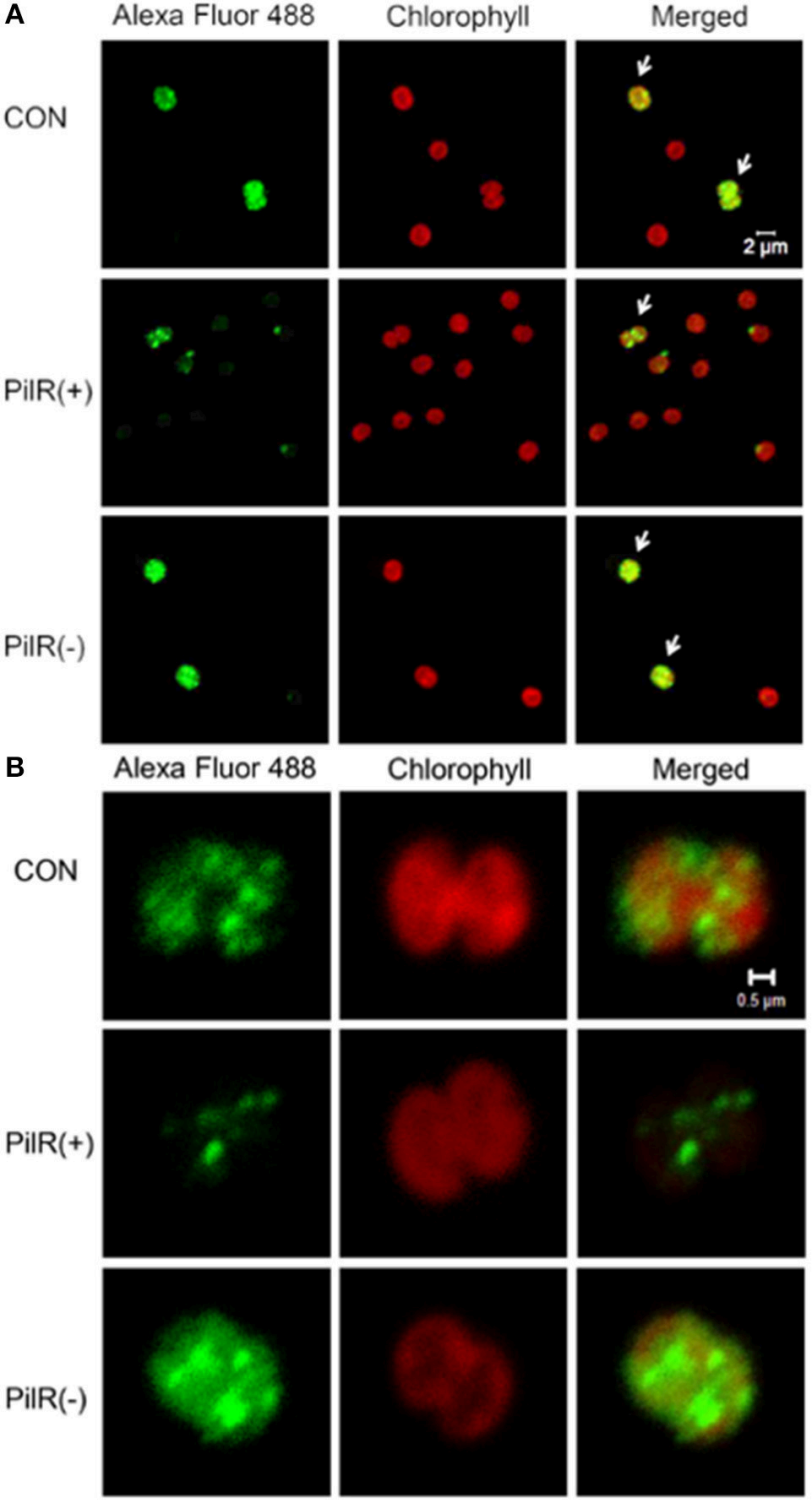
Up–regulated expression of PilA11 protein during cell division. **(A)** A group of individual cells. **(B)** A single cell. Fluorescence images of control and PilR(+)/(−) mutant cells labeled with PilA11–Alexa Fluor 488. Arrows indicate cells undergoing division. White bars = 2 and 0.5 μm.

To visualize the influence of PilR on PilA11 protein, we observed the *in vivo* localization of PilA11 protein in control, PilR(+), and PiIR(−) strains using immunofluorescence and confocal microscopy. PilR(+) cells exhibited weaker fluorescence compared to the control strain, but PilR(−) cells had significantly enhanced fluorescence compared to the control (Figures 3, 4; Table 1, ^**^P < 0.01), in agreement with their protein levels (Figure 2E). When enlarged and visualized at the single cell level, the fluorescence signal could be observed clearly at the cell surface of all strains (Figure 3), as expected given its role in cell motility. We also observed high levels of fluorescence in all three strains in dividing cells (Figure 4, Supplementary Figure S2), suggesting that PilA11 protein is enriched at the cell surface during division regardless of PilR levels.

**Table 1:** PilA11 protein fluorescence intensity in the PilR mutant cells.

**Cell state**	**CON**	**PilR(+)**	**PilR(−)**
Interphase	14, 281 ± 2, 481	5, 066 ± 1, 547^**^	22, 366 ± 3, 809^**^
Cell division	51, 911 ± 4, 661	14, 868 ± 5, 945^**^	78, 113 ± 3, 194^**^

*The fluorescence intensity of PilA11 protein was measured and calculated using the Quantity One software package, version 4.0.0 (Bio–Rad, http://www.bio-rad.com/) in 50 individual cells for each of the three strains. All values are means ± standard deviation. Significant differences between the control (CON) and test values were determined using a one–way ANOVA*.

** *P < 0.01 vs. CON*.

### Pilus formation and cell motility of the PilR mutant strains

Previous studies showed that PilA11 was an essential protein for cell motility and thick pili formation (Bhaya et al., 2001; Panichkin et al., 2006). To test the effect of PilR on PilA11 protein function, we examined cell motility and thick pili formation in the PilR strains. Cyanobacterial cells were grown under normal conditions, and cells were maintained in the presence of 20 μg·mL^−1^ kanamycin. However, for eliminating possible phenotypic alterations due to the antibiotic, the final cultures without kanamycin were used in the experiments.

**Figure 5:**
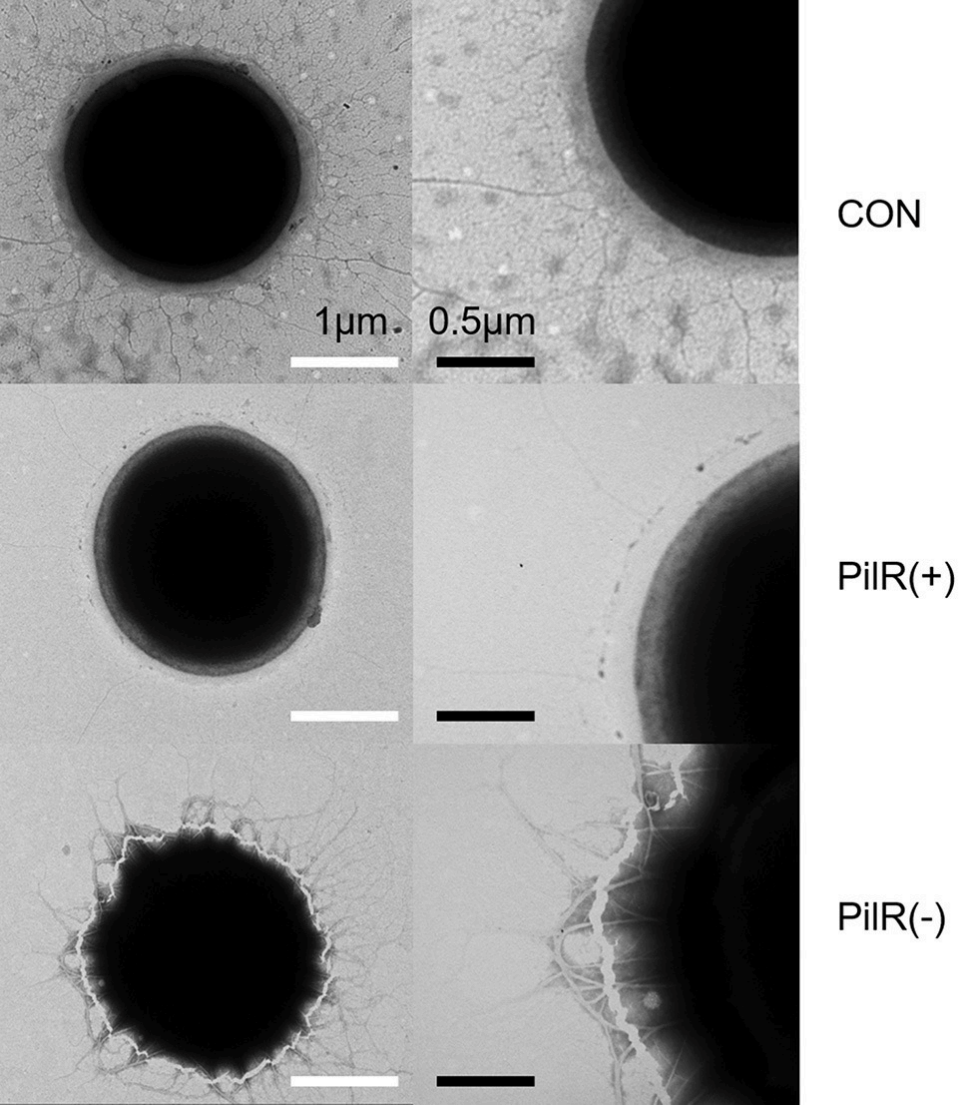
Electron micrograph of control, PilR(+), and PiIR(−) cells. Cells were processed by negative staining techniques and examined with an electron microscope. The pili of PilR(+) cells were thinner than those of the control cells, and the pili of PilR(−) cells were thicker than those of the control cells. White bars = 1 μm; black bars = 0.5 μm.

We examined control, PilR(+), and PiIR(−) cells with an electron microscope to investigate whether changes in PilR expression affected the formation of pili. As shown in Figure 5, the pili of PilR(+) cells were significantly thinner and fewer than the well-developed, normal pili of the control cells. Pili of PilR(−) cells were thicker and denser than those of the control. The number and diameter of pili of fifty individual cells each of the control, PilR(+), and PiIR(−) strains were examined and the results confirmed that PilR overexpression or suppression significantly affected pilus formation (Table 2).

**Table 2:** Number and diameter of pili in PilR mutant cells.

**Pili**	**CON**	**PilR(+)**	**PilR(−)**
Number (per cell)	36.7 ± 4.5	7.7 ± 1.5^**^	57.3 ± 4.0^**^
Diameter (nm)	4.80 ± 0.60	3.26 ± 0.30^**^	6.06 ± 0.30^**^

*Number of pili was calculated for the cross-sections of 50 cells. All values are means ± standard deviation. Significant differences between the control (CON) and test values were determined using a one-way ANOVA*.

** *P < 0.01 vs. CON*.

**Figure 6:**
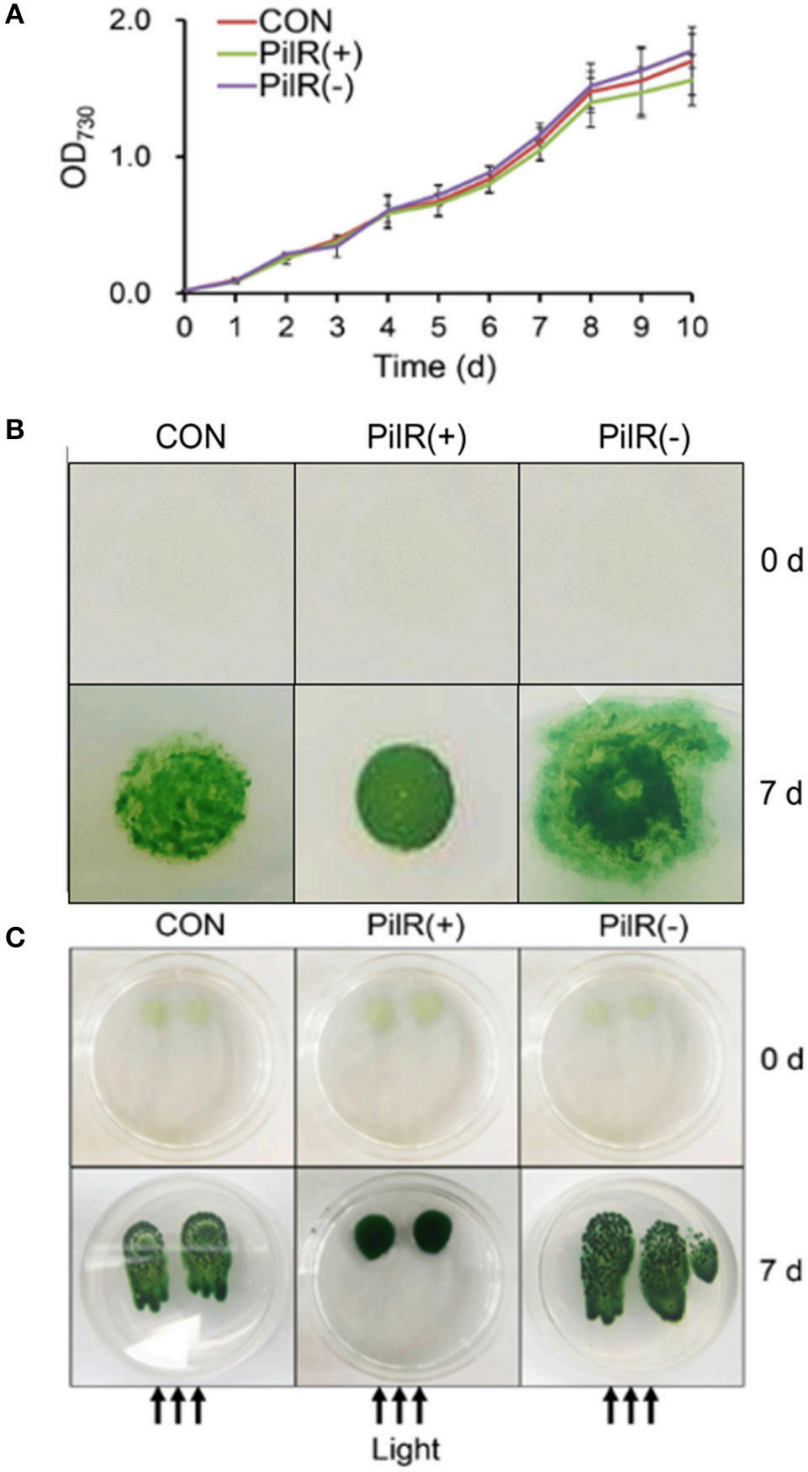
The effect of PilR on growth and motility in *Synechocystis* sp. PCC 6803. **(A)** Growth of cells in liquid culture. OD_730_, optical density at 730 nm. **(B)** Exponentially growing cells (200 μL) were spotted onto a 1% agar BG11 plate. The plates were placed under an incandescent light source of 30 μmol photons·m^−2^·s^−1^ and allowed to grow for 7 days. **(C)** Comparison of the agar surface-based phototaxis assays. Cells were applied to the surface of 0.5% agar with a micropipette and placed in front of a unidirectional light source of 30 μmol photons·m^−2^·s^−1^ for 7 days. The arrows indicate the direction of light.

To investigate the effects of PilR on cell growth and motility in *Synechocystis*, we analyzed the three strains under normal conditions in liquid culture or on agar plates. As shown in Figure 6A and Table 3, when cultured in liquid BG11, the growth rate and pigmentation were not affected in either the PilR(+) or PilR(−) strains. We examined the effects of PilR overexpression or suppression on motility by monitoring the colonies shape formed on agar plates. The colonies of the PilR(−) strain were larger and more diffuse than the control colonies, whereas those of the PilR(+) strain were smaller and more centralized (Figure 6B).

**Table 3:** The effect of PilR on pigmentation in *Synechocystis* sp. PCC 6803.

**Pigmentation**	**CON**	**PilR(+)**	**PilR(−)**
Chlorophyll *a* (mg/L)	4.09 ± 0.54	4.34 ± 0.27	4.02 ± 0.23
Carotenoid (mg/L)	1.46 ± 0.28	1.59 ± 0.16	1.37 ± 0.20

*All values are means ± standard deviation. Significant differences between the control (CON) and test values were determined using a one–way ANOVA*.

We then performed agar surface-based phototaxis assays to clarify the differences in motility between the control, PilR(+), and PiIR(−) strains (Figure 6C). When the cells were dot plated and exposed to a unidirectional light source for 7 days, the control and PilR(−) mutant strains showed positive phototactic movement, with the latter having a stronger phenotype. The PilR(+) mutant strain showed almost no sign of phototactic movement (Figure 6C), suggesting that PilR expression influences *Synechocystis* cell motility in a concentration-dependent manner.

## Discussion

Many species of cyanobacteria move by gliding, twitching, or swimming. Unlike *Escherichia coli* and *Chlamydomonas reinhardtii*, which use flagella, cyanobacteria use a pilus apparatus for motility (Waterbury et al., 1985; Häder, 1987). The oxygenic phototrophic cyanobacterium *Synechocystis* exhibits twitching motility (Stanier et al., 1971; Ng et al., 2003). Two morphologically distinct pilus types, thick and thin, exist in wild-type *Synechocystis* cells. Thick pili, possibly encoded by *pilA1*, are similar to type IV pili in many functional and morphological characteristics. Thin pili with smaller diameter are shorter than typical type IV pili (Bhaya et al., 2000). Type IV pilus biogenesis requires a complex polypeptides assemblage, located in the cytoplasmic membrane, the periplasm, or the outer membrane, for post-translational modification (e.g., PilD), assembly and export (e.g., PilC, PilQ) (Bhaya et al., 2000).. Type IV pili subunits (i.e., PilA) have a conserved, hydrophobic α-helix domain at the N-terminus, which consists of 20–25 amino acids that forms the hydrophobic pilus core (Proft and Baker, 2009). *pilA1* is responsible for the structure, motility, and transformation efficiency of thick pilus (Bhaya et al., 1999; Yoshihara et al., 2001). Mutants with disrupted *pilA2*, which encodes a second pilin-like protein, are still motile with normal cell-surface pili morphology and density. By contrast, inactivation of *pilD*, which encodes the leader peptidase, or *pilC*, which encodes a protein required for pilus assembly, abolishes cell motility and causes the absence of both pilus morphotypes (Bhaya et al., 2000). In our study, suppression of *pilA11* by overexpression of its antisense RNA PilR [PilR(+)] leads to thin, sparse pili, whereas PilR suppression [PilR(−)] has the opposite effect (Figure 5). This indicates that *pilA11* may also contribute to thick pilus biogenesis and, indeed, the ratio of thick pili to all pili was altered in the PilR mutant strains.

A locus containing five genes (*pilA9-pilA10-pilA11-slr2018-slr2019*) was discovered in *Synechocystis* in an analysis of transposon-generated mutants (Bhaya et al., 2001). The protein encoded by *pilA10* shows weak similarity to members of the PilA-like protein family (Bhaya et al., 1999), but the proteins encoded by *pilA11* and *slr2018* lack obvious functional motifs and have no clear homologs in protein databases. We observed that expression of *pilA11*, but not *pilA9, pilA10*, or *slr2018*, is negatively affected by its antisense sRNA PilR (Figure 2), and that overexpression of PilR disturbs the biogenesis of the thick pilus morphotype (Figure 5), which affects cell motility (Figure 6). These results suggest that PilR negatively regulates *pilA11* and has an important function in cell motility.

SpkA and the ATPase PilT are required for motility in *Synechocystis* (Kamei et al., 2001b; Okamoto and Ohmori, 2002). Bhaya et al. showed that the *pilA10, pilA11*, and *slr2018* genes were essential for cell motility (Bhaya et al., 2001). This study found that on 1% agar-solidified plates the *pilA11* asRNA overexpressor PilR(+) colonies are small and distinct, whereas the suppressor PilR(−) colonies are large and diffuse (Figure 6B). *spkA*::Cm^r^ mutant cells, in which the *pilA9-pilA10-pilA11-slr2018* operon is down-regulated, also form distinct colonies similar to PilR(+) (Panichkin et al., 2006). The observed phenotype of PilR(+) mutant strains is consistent with a previous analysis showing that the putative *pilA9-pilA10-pilA11-slr2018* operon in *Synechocystis* might be involved in the formation of thick pili (Panichkin et al., 2006). However, the effect that expression of the operon has on *Synechocystis* cells has not been deciphered until now.

sRNAs regulate gene expression from polycistronic messages through a variety of mechanisms (Balasubramanian and Vanderpool, 2013), which can alter expression of select genes in an operon by inhibiting the translation of genes or by altering the stability of mRNA, resulting in discoordinate regulation of the target mRNA (Møller et al., 2002; Kalamorz et al., 2007; Desnoyers et al., 2009). Alternatively, they can affect expression of all genes in an operon by sRNA-mRNA interactions, causing coordinate regulation (Rice and Vanderpool, 2011; Lu et al., 2012). The 210 nt sRNA PilR was identified as an asRNA by repressing expression of *pilA11* gene, but not *pilA9, pilA10*, or *slr2018*, in *Synechocystis* (Figure 2). It is speculated that PilR prevents *pilA11* gene expression by selective mRNA degradation. This type of regulation represses *pilA11* gene expression, but allows continued synthesis of other Type IV pilin-like proteins.

**Figure 7:**
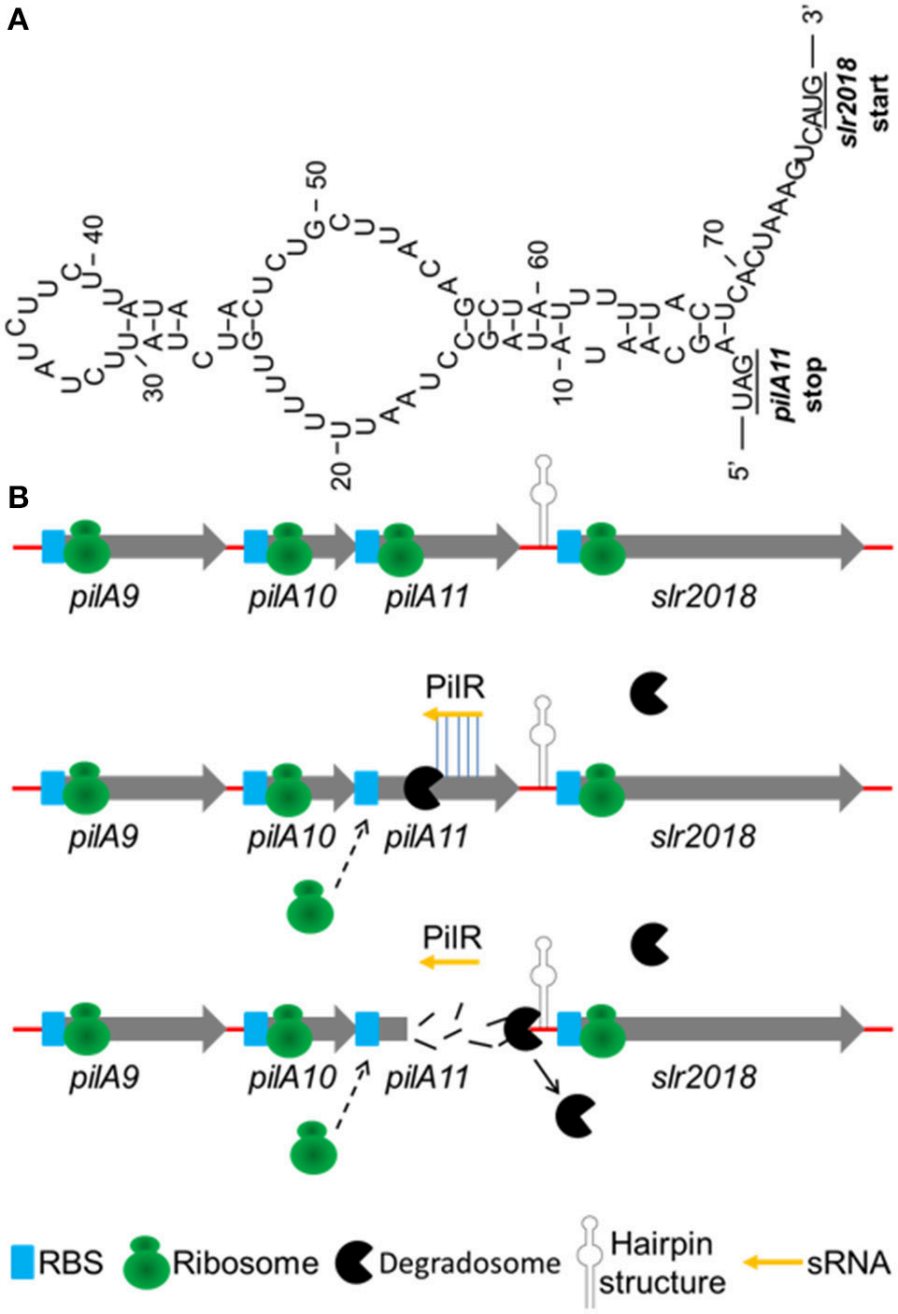
Hypothetical model on the regulation of *pilA9-pilA10*-*pilA11-slr2018* operon expression by PilR. **(A)** A secondary structure of the intergenic region between *pilA11* and *slr2018* cistrons (represented by hairpin structure in panel **B**) as determined by the mfold software (http://www.bioinfo.rpi.edu/applications/mfold/). **(B)** Normally, single-stranded *pilA9-pilA10-pilA11-slr2018* mRNA is translated into three type 4 pilin-like protein, PilA9, PilA10, PilA11 and the unknown protein Slr2018 (upper panel). While the degradation of *pilA11* mRNA is typically triggered by sRNA PilR base pairing to the target mRNA, and results in translational silencing and promoted degradosome-dependent endonucleolytic cleavage of the *pilA11* portion of the mRNA (middle panel). The degradosome is stopped and discharged by the hairpin structure between the *pilA11* and *slr2018* cistrons and thus selectively prevents the *slr2018* mRNA from degradation (lower panel).

To further elucidate the discoordinate regulation mechanism of the cis-type PilR transcript to *pilA11* gene, a closer sequence inspection is needed in both target gene *pilA11* and asRNA PilR, their cleavage by RNases. By sequence analysis, a strong secondary structure (Figure 7A) in the intergenic region between the *pilA11* and *slr2018* cistrons was predicted. Add up to the present understanding of the function of PilR in the regulation of *pilA11* gene expression, a hypothetical model on the regulation of *pilA9-pilA10-pilA11-slr2018* operon expression by PilR was proposed (Figure 7). Normally, single-stranded *pilA9-pilA10-pilA11-slr2018* mRNA is translated into three type IV pilin-like protein, PilA9, PilA10, PilA11 and the unknown protein Slr2018 (Figure 7B, upper panel). While the degradation of *pilA11* mRNA is typically triggered by sRNA PilR base pairing to the target mRNA, and results in translational silencing and promoted degradosome-dependent endonucleolytic cleavage (Kaberdin et al., 2011; Balasubramanian and Vanderpool, 2013) of the *pilA11* portion of the mRNA, presumably by RNase E (Figure 7B, middle panel). The degradosome is stopped and discharged by the hairpin structure (Figure 7A) between the *pilA11* and *slr2018* cistrons and thus selectively prevents the *slr2018* mRNA from degradation (Figure 7B, lower panel). The suggested model provides novel insights into how sRNA and the intergenic region secondary structures can work together to discoordinatly regulate target gene in an operon in *Synechocystis* (Figures 2A,E).

Cis-encoded asRNA transcripts typically regulate target gene expression either negatively, as is the case for IsrR (Dühring et al., 2006) and As1_flv4 (Eisenhut et al., 2012), or positively, as is the case for PsbA2R, PsbA3R (Sakurai et al., 2012), and RblR (Hu et al., 2017). These asRNAs are involved in various processes (Kopf and Hess, 2015), but prior to this study, no asRNAs related to cell motility were known. Our results show that the asRNA PilR is encoded by the antisense strand of the *pilA11* gene and negatively regulates its expression through complementary base pairing. Downregulation of this member of the putative *pilA9-pilA10-pilA11-slr2018* operon reduces the number and thickness of the pili, affecting cell motility. These results add a new layer of complexity to the regulatory mechanisms controlling *pilA11* gene expression and function and hence cell motility.

## Experimental procedures

### Strains and growth conditions

Wild-type *Synechocystis* was cultured as described previously (Hu et al., 2017). Solid medium supplemented with 1% (w/v) or 0.5% (w/v) agar is used to observe colony morphology for motility evaluation. After generating mutant strains (see below), kanamycin (20 μg/mL) was added to the growth medium to identify the transformed cells. Antibiotics were excluded during phenotyping to avoid interactions. The colonies were trained for 7 days under lateral illumination with a white fluorescent lamp (~30 μmol photons·m^−2^·S^−1^).

### RNA extraction and northern blot analysis

Total RNA extraction was performed as described previously (Hu et al., 2017). Northern blot analysis was performed as previously described (Hu et al., 2014). [γ-^32^P] ATP (PerkinElmer, USA) was used for the labeling of probes. DNA oligonucleotides used for the Northern blot analysis are listed in Table 4.

**Table 4:** Oligonucleotides used in this study.

**Name**	**Sequence (5′−3′)**	**Experiment**
PilR-R	TTGGAGTTACGGGAAACCTTAC	PilR probe
3′ linker	phosphorylated-AAGATGAATGCAACACTTCTGTACGACTAGAGCAC-NH_2_	RACE
3′ RTrevlinker	GTGCTCTAGTCGTACAGAAGTGTTGCATTCATC	RACE
3′ PCRrevlinker	GTGCTCTAGTCGTACAGAAGTGTTGCATTCATC	RACE
PilR-rev	TAATAATTACACTGCCGGCGG	5′ RACE, first PCR
PilR-rev2	ACGGGGCTATCGCCTCAA	5′ RACE, second PCR
PilR-rev3	GCAAGCAACTATTGATGGGGT	5′ RACE, third PCR
PilR-fw1	GGAAGAAGTCTAGTGCAATCGGA	3′ RACE, first PCR
PilR-fw2	ATTGAGGCGATAGCCCCGTTC	3′ RACE, second PCR
PilR-qRT-F	GAAGTCTAGTGCAATCGGAAGG	qRT-PCR
PilR-qRT-R	TTGGAGTTACGGGAAACCTTAC	qRT-PCR
pilA9-qRT-F	TAGTCGTGGTGGTGATTGGC	qRT-PCR
pilA9-qRT-R	TGCCCTAGTTCTAGCGGTCT	qRT-PCR
pilA10-qRT-F	TGAGTTGGACGCCCAGTTAG	qRT-PCR
pilA10-qRT-R	GGAACAAGTCCCGTTGGGAT	qRT-PCR
pilA11-qRT-F	CTGAAATTCCTCCCGCTGGT	qRT-PCR
pilA11-qRT-R	TGCCCATTGCCGTTGTAGAT	qRT-PCR
slr2018-qRT-F	TCTCCGGTTTGTTGGTAGCC	qRT-PCR
slr2018-qRT-R	ATTCAGGGCTCCTTCTGCAC	qRT-PCR
5′*rnpB*	AATGCGGTCCAATACCTCC	Mutagenesis (overlap extension PCR)
3′*rnpB*/*kana*	GTTACCCA TGATATCTCTTTTTCTAGTGTGCCATTG	Mutagenesis (overlap extension PCR)
5′*kana*/*rnpB*	CTAGAAAAAGAGATATCAGTTGGGTAACGCCAGGG	Mutagenesis (overlap extension PCR)
3′*kana*	CACTTTATGCTTCCGGCTCG	Mutagenesis (overlap extension PCR)
*slr0168*-F	ACCTCTCCACGCTGAATTAGA	Mutagenesis (*slr0168*, upstream)
*slr0168*-R	TAATACCCACCGCACTGACC	Mutagenesis (*slr0168*, downstream)
PilR(+)-F	GAAGTCTAGTGCAATCGGAAGG	Mutagenesis (PilR, upstream)
PilR(+)-R	TTGGAGTTACGGGAAACCTTAC	Mutagenesis (PilR, downstream)
PilR(−)-F	TAGAAGAACGGGGCTATCGC	Mutagenesis (anti-PilR, upstream)
PilR(−)/*oop* ter-R	GGAATAAAAAACGCCCGGCGGCAACCGAGCGTTGAAGTCTAGTGCAATCGGAAGG	Mutagenesis (anti-PilR, downstream)
0168-F	CCCTGAAGTTAGCCAGTTTAATTG	PCR
0168-R	GTCACTGAAGCGGTCTAACTTAGC	PCR

*The 3′ RACE analysis was performed prior to 5′ RACE. All PCR primers for 5′ RACE were designed according to the results of 3′ RACE. fw, forward; rev, reverse*.

### 5′- and 3′- rapid amplification of cDNA ends (RACE)

The 5′ end and 3′ end RACE was performed according to Hu et al. (2014). All oligonucleotides and primers used in the RACE analysis are listed in Table 4.

### qRT-PCR validation

The qRT-PCR analysis was done by standard procedure using cDNA as previously described (Hu et al., 2014, 2017). All data are shown as the mean ± *SD* (*n* = 5). All primers used for the analysis are listed in Table 4.

### Mutagenesis

Mutant strains PilR(+), and PilR(−) were created as previously described (Golden et al., 1987; Hu et al., 2017). All primers used for this analysis are listed in Table 4.

### Protein gel and immunoblot analysis

Protein gel and immunoblot analysis were performed as previously described (Hu et al., 2014). The membranes were probed with rabbit primary anti-PilA11 antibodies (1:5,000; QWbio, http://www.qwbio.com Beijing).

### Electron microscopy

Specimens were prepared for electron microscopy using the conventional negative staining procedure (Bhaya et al., 1999) with the following modifications. Briefly, a 200 μL drop of sample solution was adsorbed onto a glow-discharged carbon-coated copper grid for 10 min, stained with two drops of freshly prepared 2% phosphotungstic acid (pH 7.0), and examined using a Hitachi HT7700 microscope.

### Immunofluorescence microscopy

Microscopy analysis of cells grown in liquid BG11 medium was carried out using a confocal scanning microscope (Leica TCS SP8, Wetzlar, Germany). PilA11 localization was performed as previously described (Miyagishima et al., 2005; Zhan et al., 2016). The fluorescence intensity was measured and quantified using the Quantity One software package, version 4.0.0 (Bio-Rad, http://www.bio-rad.com/) in 50 individual cells for each of the three strains.

### Measurements of chlorophyll *a* and carotenoid contents

Chlorophyll a and carotenoid contents were measured as described (Zhang et al., 2013). Chlorophyll *a* (C*a*) and total carotenoid (Cc) contents were calculated according to Equation (1) (Harmut and Lichtenthaler, 1987).

(1) $$\begin{array}{l} \text{C}a=16.72{\text{A}}_{665.2}-9.16{\text{A}}_{652.4}\\ \text{Cc}=\left(1000{\text{A}}_{470}-1.63\text{C}a\right)/221 \end{array}$$

### Calculating the number and diameter of pili

Number and diameter of pili were calculated for the cross-sections of 50 cells of electron microscopy using ImageJ software. All values are means ± standard deviation. Significant differences between the control (CON) and test values were determined using a one-way ANOVA. ^**^P < 0.01 vs. CON.

## Author contributions

JH and QW: Designed the study; JH, JZ, HuiC, CH, and QW: Collected, analyzed, and interpreted the data; JH, HuaC, and QW: Wrote the manuscript.

### Conflict of interest statement

The authors declare that the research was conducted in the absence of any commercial or financial relationships that could be construed as a potential conflict of interest.
