# Supplementary material for: The Small Regulatory Antisense RNA PilR Affects Pilus Formation and Cell Motility by Negatively Regulating pilA11 in Synechocystis sp. PCC 6803
Source: Front Microbiol. 2018 Apr 23;9:786. doi: 10.3389/fmicb.2018.00786 (PMC5924778; doi:10.3389/fmicb.2018.00786)
Supplement: Supplementary file 1 [file Image1.PDF]

Supplementary Information

The small regulatory antisense RNA PilR affects pilus formation and cell motility by negatively regulating *pilA11* in *Synechocystis* sp. PCC 6803

Jinlu Hu[1], Jiao Zhan[2], Hui Chen[2], Chenliu He[2], Huaixing Cang[1]，and Qiang Wang[2,*]

[1] School of Life Sciences, Northwestern Polytechnical University, Xi'an, Shanxi 710072, China
[2] Key Laboratory of Algal Biology, Institute of Hydrobiology, the Chinese Academy of Sciences, Wuhan, Hubei 430072, China

[*] *Corresponding author*
E-mail: wangqiang@ihb.ac.cn, Tel: +86-27-68780790, Fax: +86-27-68780123

Running title
PilR negatively regulates *PilA11* and cell motility

Key words
Synechocystis sp. PCC 6803, PilR, *pilA11*, sRNA, cell motility

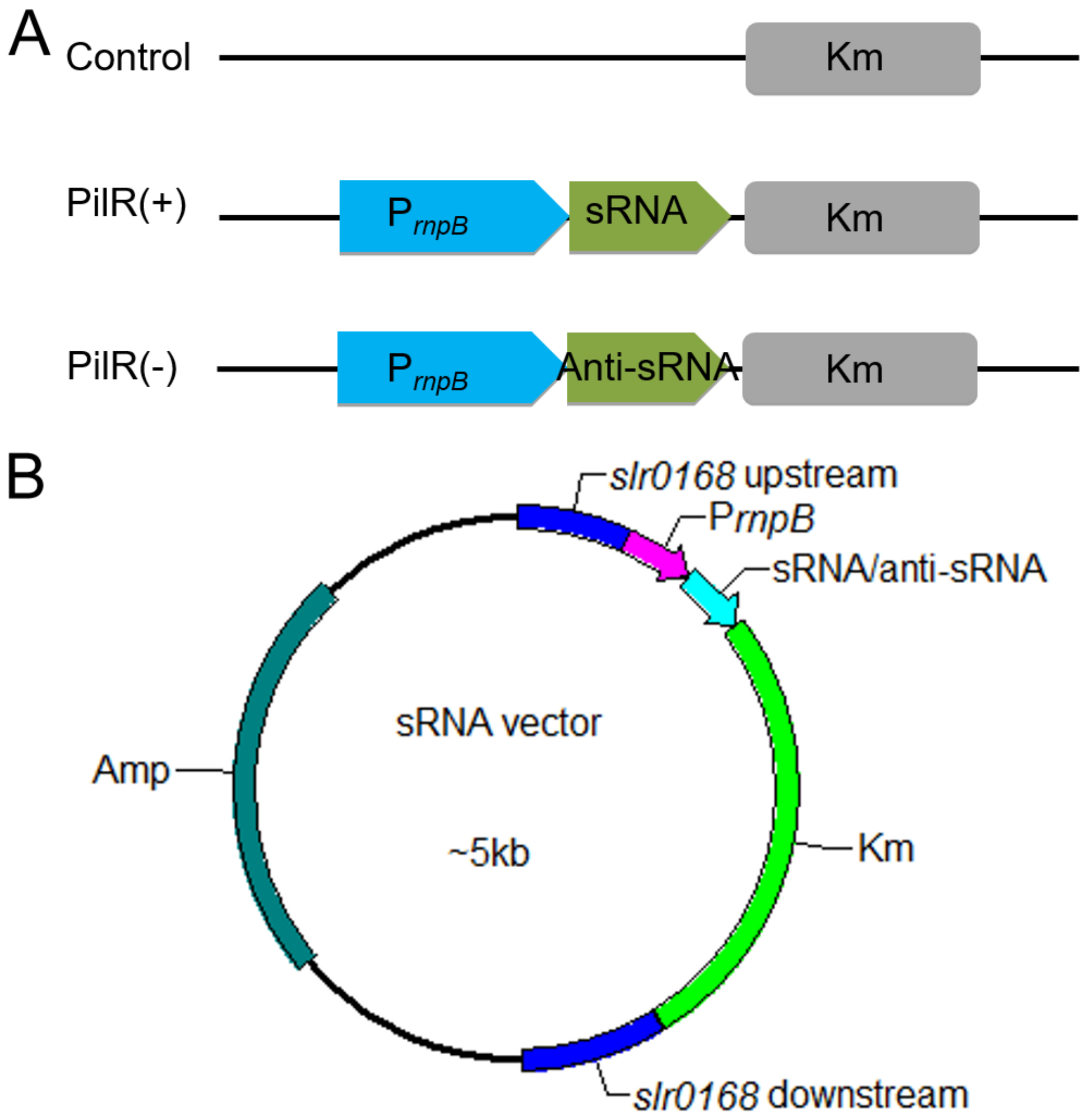

Supplementary Figure S1. Overexpression and suppression of PilR. (a) The sense and antisense fragments of PilR were fused to the *rnpB* promotor, yielding overexpressor PilR(+) and suppressor PilR(-), respectively. The control strain contains only the *kanamycin* resistance cassette. (b) Schematic diagram of PilR(+)/(-) mutant construction.

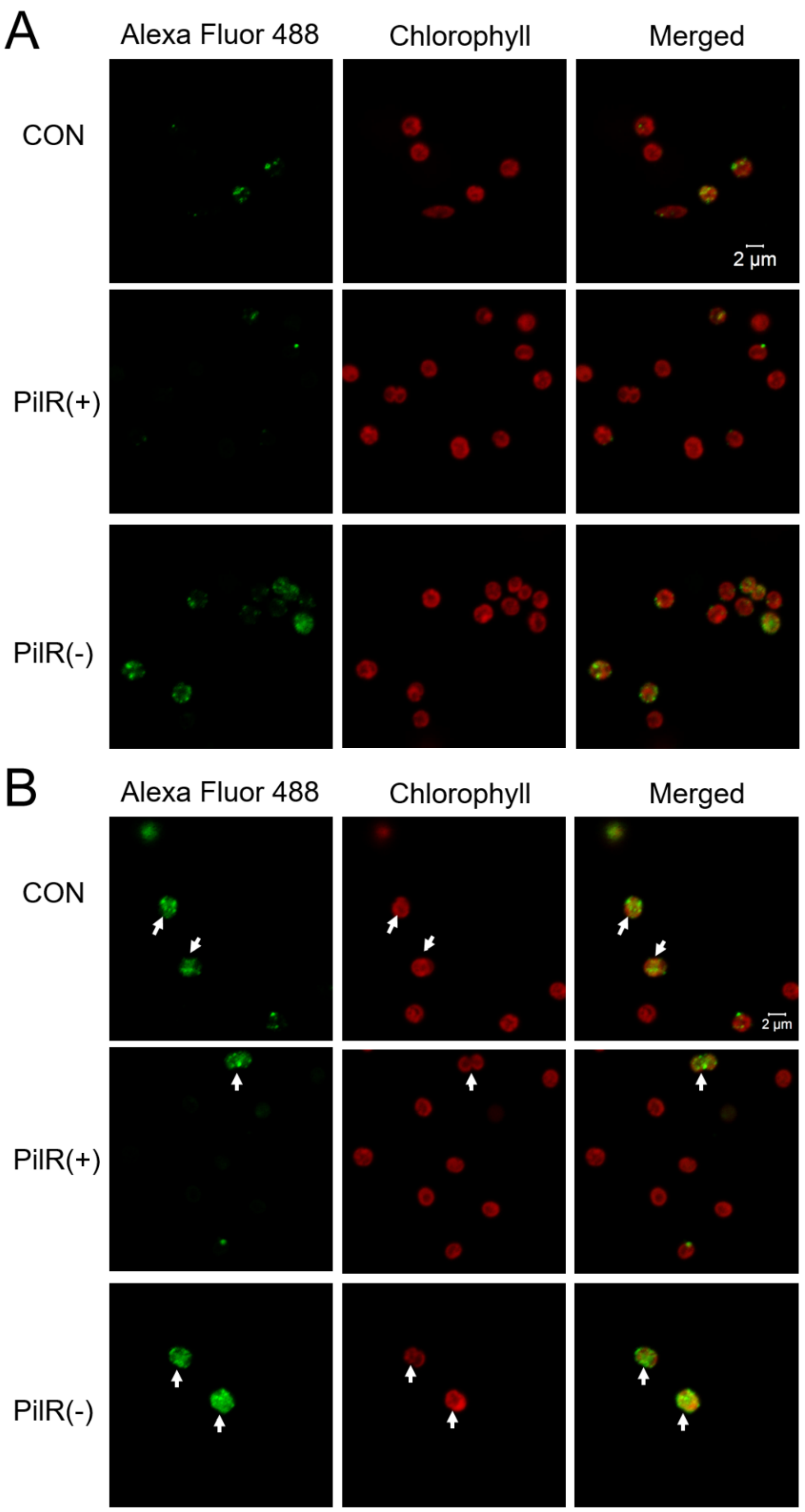

Supplementary Figure S2. Fluorescence images (showing cell population) of control and PilR(+)/(-) mutant cells labeled with PilA11-Alexa Fluor 488. (A) Cells during interphase recording. (B) Cells division recording. Merged indicates that the images of cells labeled with Alexa Fluor 488 and chlorophyll are merged and shown in green and red, respectively. Arrows indicate cells undergoing division. White bars = 2 µm.
